# Hindgut Duplication: A Unique Case of Six Perineal Openings

**DOI:** 10.7759/cureus.1433

**Published:** 2017-07-06

**Authors:** Shujaul Haq, Adeel Nasrullah, Iftikhar Ahmed, Haider Ghazanfar, Abu Baker Sheikh, Aisha Akhtar

**Affiliations:** 1 Department of Internal Medicine, Shifa International Hospital; 2 Department of Pediatric Surgery, Military Hospital Rawalpindi, Pakistan; 3 Internal Medicine, Newark Beth Israel Medical Center; 4 Surgery, Texas Tech Health Sciences Center Lubbock

**Keywords:** hindgut, genitourinary, complete duplication, diagnostic imaging

## Abstract

Complete hindgut duplication is a rare and intriguing entity, often coupled with genitourinary abnormalities and neural tube defects. The diagnosis demands a thorough clinical exam and radiological workup. Timely recognition and expeditious treatment of these patients can lead to a better quality of life. We present a case of a 10-month-old female with complete hindgut duplication and associated genitourinary duplication treated with surgical intervention.

## Introduction

Gastrointestinal (GI) duplications are a rare occurrence and may involve any part of the alimentary tract. The diverse clinical presentations depend on the size, site, and type of duplication. Complete hindgut duplication, an extremely rare condition, often presents with associated genitourinary duplications.

We present a case of total duplication of the hindgut. The patient also had a duplication of the genitourinary system. A review of the literature revealed only a handful of patients with the same condition. One case reported by Schatz had a complete duplication of bladder, urethra, vagina, uterus, and anus [1]. The management of duplications varies greatly according to the anomalous anatomy. If surgical management is not undertaken, a possibility of neoplastic change should not be ruled out [2]. What makes our case more unique is that our patient had an associated situs inversus, which to the best of our knowledge, has not been reported with any case of hindgut and genitourinary duplication. The purpose of our study is to identify this rare set of associated conditions early because prompt surgical treatment has shown a favorable prognosis and better quality of life for patients. Informed consent statement was obtained for this study.

## Case presentation

A 10-month-old female born at term was brought to the outpatient department by her mother with a complaint of fecal discharge from the vagina since day twenty of birth and constipation with mild abdominal distention for the last two months. The mother had noted fecal discharge from the normal anal opening as well as the vagina. Past medical, surgical and birth history were unremarkable. On examination, the patient was lying comfortably and was responsive to her surroundings. Her vital signs were stable. Abdominal examination revealed soft abdomen with mild distention. There was no tenderness or any palpable mass and no signs of ascites. Bowel sounds were audible. Digital rectal examination (DRE) showed normal anal opening. On rectal exam, a soft bulge was felt on the anterior wall of the rectum with an impression of an extrinsic growth. There was a fistulous opening in the vestibule of the vagina from which fecal matter was exuding. Rest of the systemic examination revealed no abnormalities.

**Figure 1:**
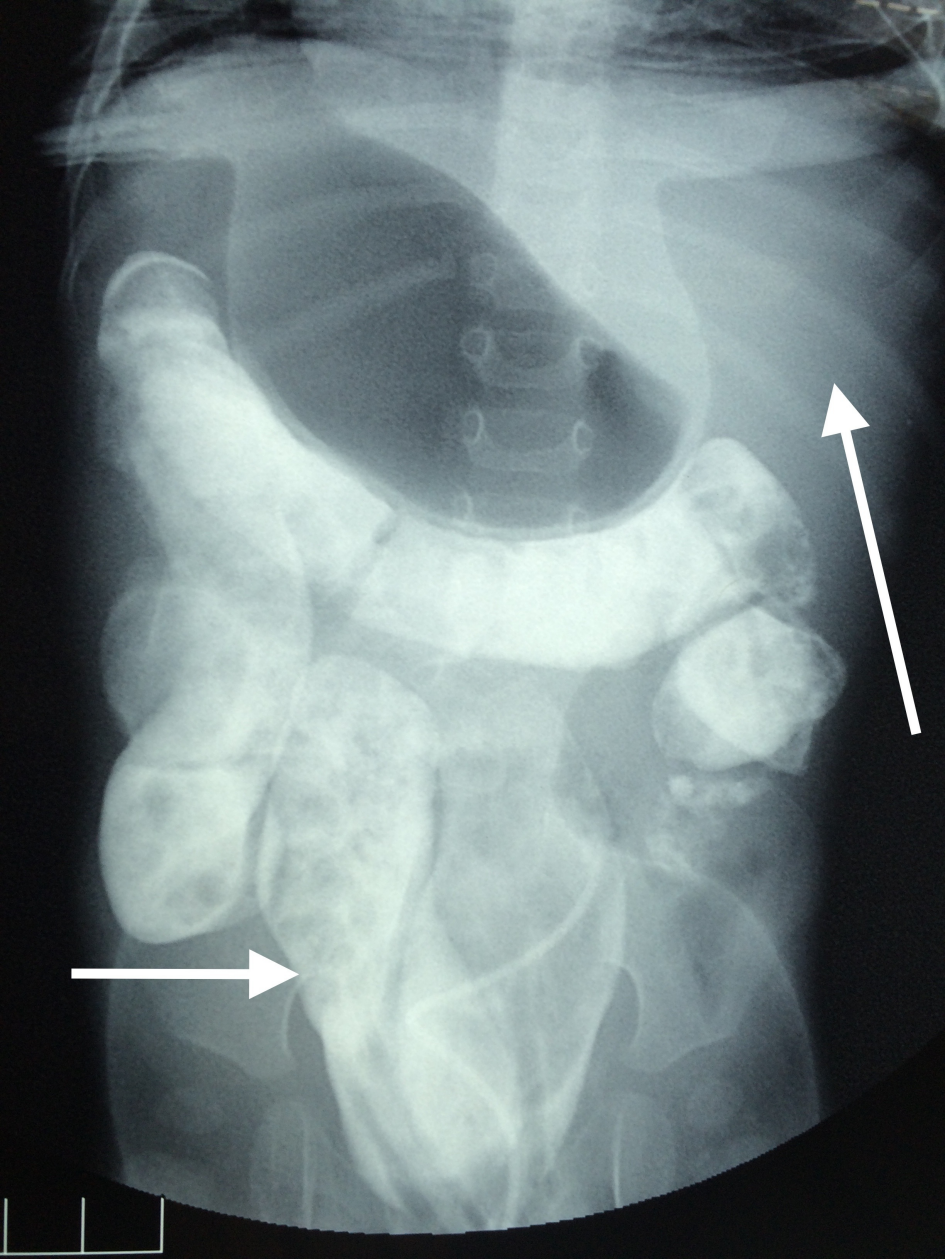
Contrast enema abdominal radiograph showing duplicated sigmoid colon (small arrow), liver on the left side (large arrow)

Routine laboratory investigations were normal. Contrast enema revealed duplication of the anal canal, rectum, sigmoid colon and situs inversus with liver on the left as shown in Figure 1.

**Figure 2:**
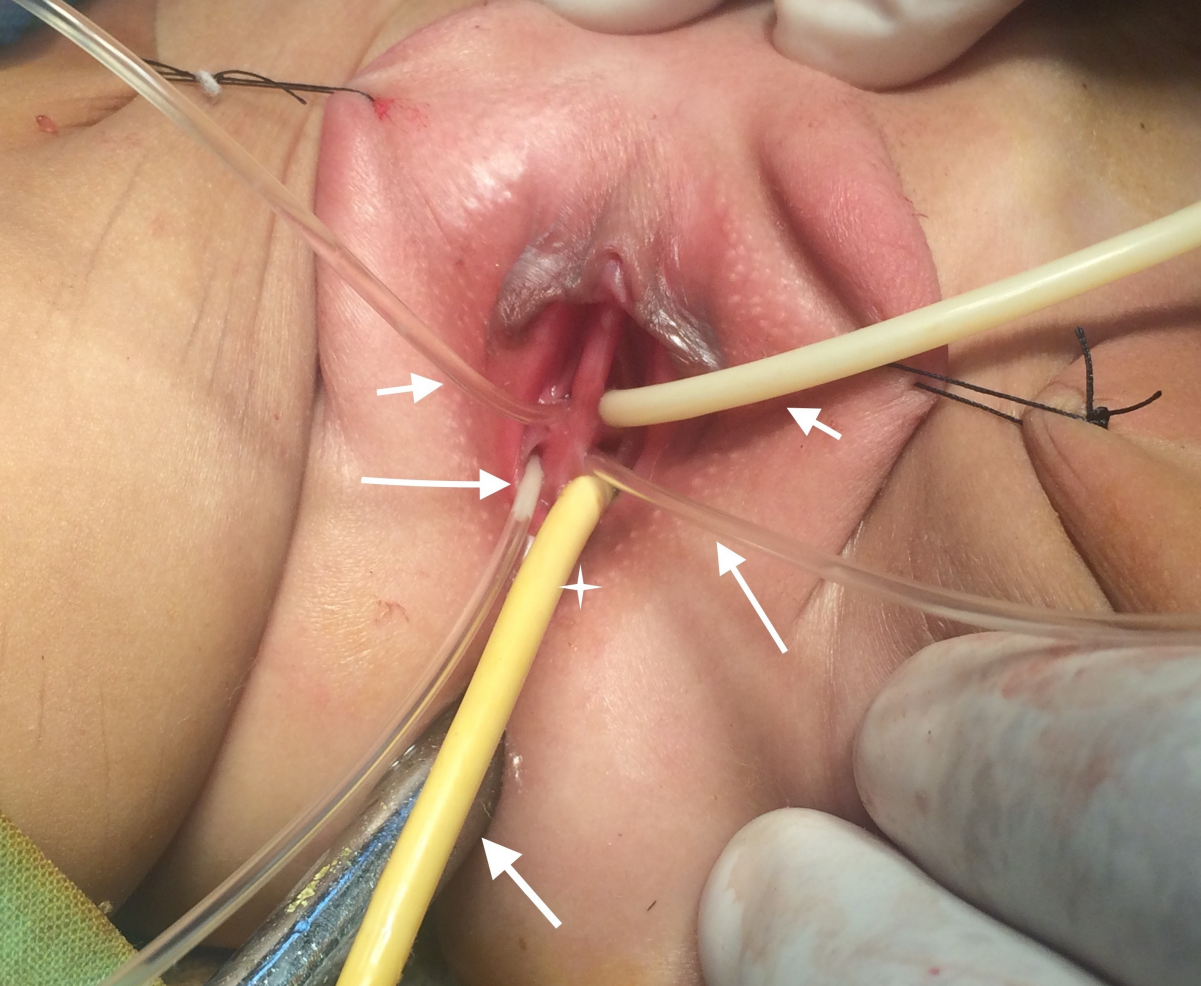
Examination under anesthesia showing two urethral openings (small arrows at the top), two vaginal openings (large arrows), ectopic anal opening (star), and normal anal opening at the bottom (arrow below)

On the basis of history, physical examination and investigations, a diagnosis of hindgut duplication were made and examination under anesthesia (EUA) and exploratory laparotomy (EL) was planned. During EUA, six perineal openings (duplicated vagina, urethra and anus) were revealed as shown in Figure 2.

**Figure 3:**
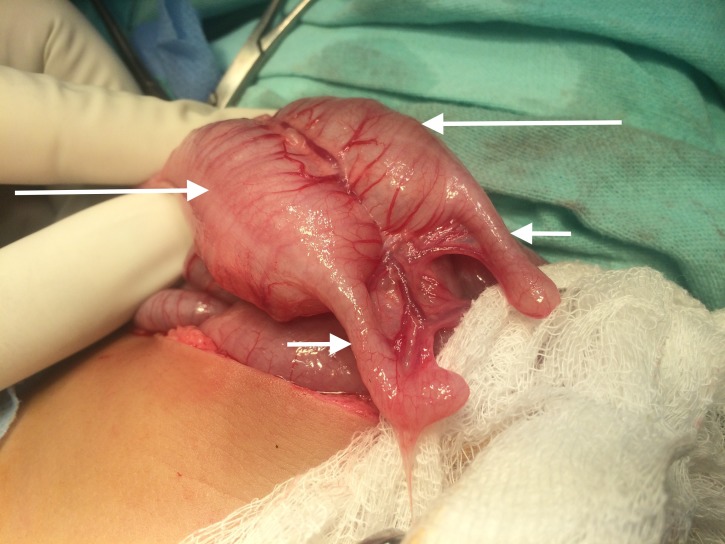
Exploratory laparotomy showing duplicated cecum (large arrows) and appendix (small arrows)

On contrast studies two urinary bladders and two vaginas were visible. EL revealed duplication of hindgut from anus till cecum/appendix as seen in Figure 3.

Distally, the ectopic anus was excised and proximally the duplicated gut was internally drained into the normal rectum. The septum between the two rectums was divided with linear cutter stapling device to create one single sigmoid and rectum. The septum between the two bladders was divided and converted to a single lumen viscera. The patient had an uneventful postoperative period and was discharged on the fifth postoperative day with a follow-up plan of care.

The repair of the double urethra was postponed till the age of three years where the urine continence status of the patient will determine further management. The management of double vagina was postponed till puberty after assessing the menstrual and fertility status of the patient.

## Discussion

Gastrointestinal (GI) duplications are a very uncommon, but complex set of embryological disorders can involve any part of the alimentary canal from the tongue to the anus. Duplications of any kind share a few common characteristics. They are hollow, lined with gastrointestinal tract epithelium and have a smooth muscle wall.

GI duplications were initially believed to be more common in males as compared to females but it was later confirmed by a study that there is an equal male to female ratio [1]. Colonic duplications account for 15% [3] and rectal duplications for 5% [4]. The exact pathogenesis of duplications is unknown and multiple theories have been put forward. According to the most credible theory, as the rapidly growing endothelial cells occlude the intestinal lumen, vacuoles form inside the cell masses due to the growth of the intestine. These vacuoles fuse to create a single lumen intestine. If one of these vacuoles pinches off, they can create a secondary lumen, which may entirely be separated from the main lumen but grows in proportion to the main lumen [5].

Colonic duplications can be cystic or tubular involving a limited portion of the colon or be extensive. They are usually divided into two types, type I and II. Type I usually has partial involvement of the colon or rectum while type II has a wider spectrum as in addition to colon and rectum, there can also be associated congenital anomalies including duplication of the lower genitourinary tract, double appendices, situs inversus and neural tube defects [5].

The presentation of this condition might vary greatly from patient to patient depending upon the degree of and site of duplication. In a colonic or anal duplication, the patients are usually asymptomatic. If symptomatic, a colonic duplication can cause symptoms related to an obstruction such as abdominal pain, distention, and constipation. There can be concurrent fistulas in tubular colonic duplications with other surrounding structures or hollow viscera such as vagina, vulva, perineum and urinary tract. Without a fistula, there can be a distention of the duplicated gut distally. In our patient, there was a presence of a recto-vaginal fistula, which presented as fecal discharge from the vagina initially. The patient also had mild progressive abdominal distention afterward.

Urogenital duplication can present as a separate entity or in association with hindgut duplication. The constellation of these associated abnormalities can be explained by "partial twinning" of the caudal end of the embryo or abnormal development of urorectal septum [6]. Duplication can either be complete or partial and can occur in any part of the urogenital tract including bladder, urethra, and vagina. Although duplication may occur in the sagittal or coronal plane, it is more commonly seen in the former. The anatomy of urogenital duplications determines if a patient presents earlier or later in life. With earlier presentations, urinary tract infections, obstructions, and voiding difficulties are usually seen. With later presentations, the patients remain largely asymptomatic and occasionally present with infertility [7-8].

Various diagnostic modalities are available for the diagnosis of GI duplications. Ultrasonography and contrast studies are most widely used. Computed tomography (CT) and magnetic resonance imaging (MRI), although less often used, are helpful in localizing and diagnosing complex duplications [9]. Treatment of duplications largely depends on whether or not a patient is symptomatic. Generally, a conservative approach is followed if no symptoms are seen. If surgery is considered, the primary objective is to treat the patient's symptoms rather than correct the anatomical abnormality. The choice of surgery depends upon two important factors. Firstly, care should be taken not to affect the blood supply of the normal bowel as the duplicated segment might share its blood supply with the normal bowel segment. Secondly, the internal drainage of the duplicated segment containing ectopic gastric mucosa can lead to gastrointestinal bleeding. Cystic duplications are easier to excise because their site of involvement is limited while tubular duplications need as extensive resection [10].

## Conclusions

Although extremely rare, hindgut duplications, if found, should be investigated and a clinical-radiological correlation should be made to accurately delineate the site and extent of the disease. Also, one must be vigilant, as these duplications are often associated with other systemic anomalies. Management of the duplication depends upon the extent and symptomatology of the disease, as larger and symptomatic duplications require more aggressive surgical management.
